# Schizophrenia and OCD, association and treatment. Literature review

**DOI:** 10.1192/j.eurpsy.2023.1422

**Published:** 2023-07-19

**Authors:** A. Tbatou

**Affiliations:** PSYCHIATRY, CHU SOUSS MASSA, AGADIR, Morocco

## Abstract

**Introduction:**

Worldwide epidemiological studies estimate that around 1% of the world population suffers from schizophrenia and 2 to 3% from compulsive-obsessive disorder (OCD). Moreover, a significant percentage of schizophrenia patients present an OCD comorbidity.

These statistics testify to a close relationship between the two pathologies, which sometimes causes difficulties in differentiating between the two diagnoses, complicated by the fact that many schizophrenia patients can suffer from obsessive and/or compulsive symptomatology similar to OCD.

**Objectives:**

This literature review aims to explore the frequency of association between schizophrenia and OCD and the administrated treatment through articles exploration.

**Methods:**

We conducted a literature review about the association frequency between schizophrenia and OCD and the administrated treatment, which implies scientific literature exploration and selecting several articles treating this topic.

For our review, keywords used to search in Scopus and PubMed were: “schizophrenia and OCD” and “schizophrenia and OCD treatment” found more than 1500 results between 1988 and 2022. With the application of exclusion criteria, we included approximately 40 recent articles treating the frequency of association between schizophrenia and OCD and administrated treatment.

We organized These articles by counties, association frequency, and the administrated treatment.

**Results:**

Our review of these articles and studies finds mostly a percentage between 10% to 30 % worldwide (countries from Asia, America, Africa, and Europe) of schizophrenia patients suffering also from OCD. The difficulty of diagnosis between schizophrenia and OCD, and the frequency of existence of obsessive and/or compulsive symptoms in schizophrenia remains one of the most relevant challenges for diagnosis and treatment in these countries.

We also found that administrated treatment was commonly pharmacological with psychotherapy association sometimes.

**Conclusions:**

Our review explored the frequency of schizophrenia and OCD association and the administrated treatment. We found significant comorbidity between the two pathologies. With these findings, we may suggest systematic research for OCD with adapted scales in every schizophrenia case.

**Disclosure of Interest:**

None Declared

